# Raman-Deuterium Isotope Probing for *in-situ* identification of antimicrobial resistant bacteria in Thames River

**DOI:** 10.1038/s41598-017-16898-x

**Published:** 2017-11-30

**Authors:** Yizhi Song, Li Cui, José Ángel Siles López, Jiabao Xu, Yong-Guan Zhu, Ian P. Thompson, Wei E. Huang

**Affiliations:** 1 Department of Engineering Science, University of Oxford, Parks Road, OX1 3PJ Oxford, United Kingdom; 2 Key Lab of Urban Environment and Health, Institute of Urban Environment, Chinese Academy of Sciences, Xiamen, 361021 China; 3 Chemical Engineering Department, University of Córdoba, Campus Universitario de Rabanales, Ctra. N-IV, km 396, building Marie Curie (C-3), CP/14071, Córdoba, Spain

## Abstract

The emergence and widespread distribution of antimicrobial resistant (AMR) bacteria has led to an increasing concern with respect to potential environmental and public health risks. Culture-independent and rapid identification of AMR bacteria *in-situ* in complex environments is important in understanding the role of viable but non-culturable and antibiotic persistent bacteria and in revealing potential pathogens without waiting for colony formation. In this study, a culture-independent and non-destructive phenotyping approach, so called Raman Deuterium Stable Isotope Probing (Raman-DIP), was developed to identify AMR bacteria in the River Thames. It is demonstrated that Raman-DIP was able to accurately identify resistant and susceptible bacteria within 24 hours. The work shows that, in the River Thames, the majority of the bacteria (76 ± 2%) were metabolically active, whilst AMR bacteria to carbenicillin, kanamycin and both two antibiotics were 35 ± 5%, 28 ± 3%, 25 ± 1% of the total bacterial population respectively. Raman activated cell ejection (RACE) was applied to isolate single AMR bacteria for the first time, linking AMR phenotype (reistance to antibiotics) and genotype (DNA sequence). The sequences of the RACE sorted cells indicate that they were potential human pathogens *Aeromonas* sp., *Stenotrophomonas* sp. and an unculturable bacterium. This work demonstrates Raman-DIP and RACE are effective culture-independent approach for rapid identification of AMR bacteria at the single cell level in their natural conditions.

## Introduction

The persistence and spread of antibiotic resistance in bacterial pathogens represents a considerable public health concern. AMR superbug infection accounts for 700,000 deaths annually and it has been estimated that the death toll could rise to one person every three seconds, which equates to 10 million people per year by 2050 if AMR is not controlled^1^. Today, many first-generation drugs are, if not all, ineffective. The paradox of antibiotics is that through their use, they not only inhibit an infection but also select for the emergence and spread of resistance, directly reducing their long-term efficacy^2^. The crisis of AMR has been attributed to the overuse and misuse for medication, as well as a lack of new drug development by the pharmaceutical industry^3^. Furthermore, an estimated 80% of antibiotics sold in the U.S. are used in animals. Up to 90% of the antibiotics given to livestock are excreted in urine and stool, then widely dispersed through fertilizer, groundwater, and surface runoff^3^. The release of antibiotics to environment also affects the environmental microbial community which form a natural pool for AMR bacteria and poses a huge risk to human health. Therefore, the investigation of the abundance and distribution of AMR bacteria in environment plays critical role in combating AMR.

To date, most studies of environmental AMR bacteria have relied on two approaches. The first approach is conventional cultivation method for obtaining AMR isolates from the community and subsequently studying their response to changing environment factors^4–8^, and the second approach is metagenomic method coupled with antimicrobial resistance gene (ARG) identification^9–13^. However, both approaches have their drawbacks. The cultivation method neglects the viable but nonculturable microbes (VBNC)^14,15^, and furthermore, it is ineffective characterising the status and activity of cells *in situ*. By contrast, metagenomics is a powerful cultivation-independent approach for studying microbes. However, it is difficult to establish a link between the genotypic ARG and the phenotypic AMR. On the one hand, possessing ARG does not necessarily lead to gene expression. On the other hand, genes conferring a resistant phenotype when expressed, are probably not “true” resistance genes that can be identified or predicted from ARG data base^16^.

Raman micro-spectroscopy is a label-free and non-destructive biochemical fingerprint technology^17–19^. It is able to provide intrinsic ‘phenotypic profile’ of single cells^17^, revealing gene expression^20^, biosynthesis of compounds^21^, cell specific components^22^, characteristic structures, physiological states^23^, or metabolic switching^14,18,24,25^. Recently, a universal Raman biomarker, C-D band around 2040–2300 cm^−1^, has been discovered to probe general metabolic activity of cells in a complex microbial community by simply adding heavy water (D_2_O) into cell cultures^26^. It is proposed that the microorganisms that are metabolically active should incorporate the deuterium to the cells via NADH/NADPH electron transport chain, leading newly formed carbon-deuterium (C-D) bond to produce a distinctive Raman band in single cell Raman spectra (SCRS)^14,25,26^.

We hypothesise that when the bacteria are exposed to the antimicrobial agents, metabolically active cells are AMR bacteria, whilst the susceptible strains are inactive due to the inhibition and killing effect of the agents. Raman-Deuterium Isotope Probing (Raman-DIP) should be able to study AMR bacteria at the single cell level, by adding heavy water into samples containing the microbial community. The resistant and susceptible population can be distinguished by examining the C-D Raman band in their SCRS. After testing this hypothesis on culturable *E. coli* bacteria and two isolates from Thames River, Raman-DIP was then applied to investigate the kanamycin and carbenicillin resistant bacteria in water samples collected from Thames River. The Raman Activated Cell Ejection (RACE)^27^ was employed to link AMR phenotype and genotype (16S-rRNA sequencing). It demonstrates that Raman-DIP should be a powerful culture-independent approach for rapid and quantitative assessment of AMR bacteria *in situ*.

## Results

### AMR identification by conventional cultivation

Samples from River Thames were plated on LB agar plates with various antibiotics. The colony forming unit (CFU) counts per ml of river water on each medium was plotted and represented in Fig. S1. The results demonstrated that a large proportion of the culturable bacteria in River Thames were antibiotic resistant, of which 23%, 67% and 3% of the total culturable bacteria respectively resisted to carbenicillin, kanamycin and both two antibiotics at the concentrations of 10 × MIC. Eight isolates were randomly picked from LB agar plates containing antibiotics, and their identities based on 16S-rRNA were shown in Table 1, which were 5 different Gram-negative strains, all belonged to the class of γ-proteobacteria.

**Table 1:** The identification of 8 AMR isolates from the Thames according to 16S-rRNA.

	Identification	Resistance	Potential as pathogen	References
Carb01/02	*Aeromonas sp*.	carbenicillin	Associated with human diseases	28
Carb03	*Pseudomonas veronii*	carbenicillin		
Carb04	*Pseudomonas putida*	carbenicillin		
Km01/03	*Citrobacter freundii*	kanamycin	Pathogen/opportunistic pathogen.	37–40
KM02/04	*Stenotrophomonas maltophilia*	kanamycin	Human pathogen	29–32

The total bacteria number in water samples from The River Thames was 5.0 × 10^6^ cells/ml, estimated by microscopic counting. The cultivation method described above only recovered 1% of the bacteria present, which were able to grow on LB agar or agar medium made by filter-sterilised river water at room temperature.

### Raman-DIP of bacterial pure culture exposed to antibiotics

**Figure 1:**
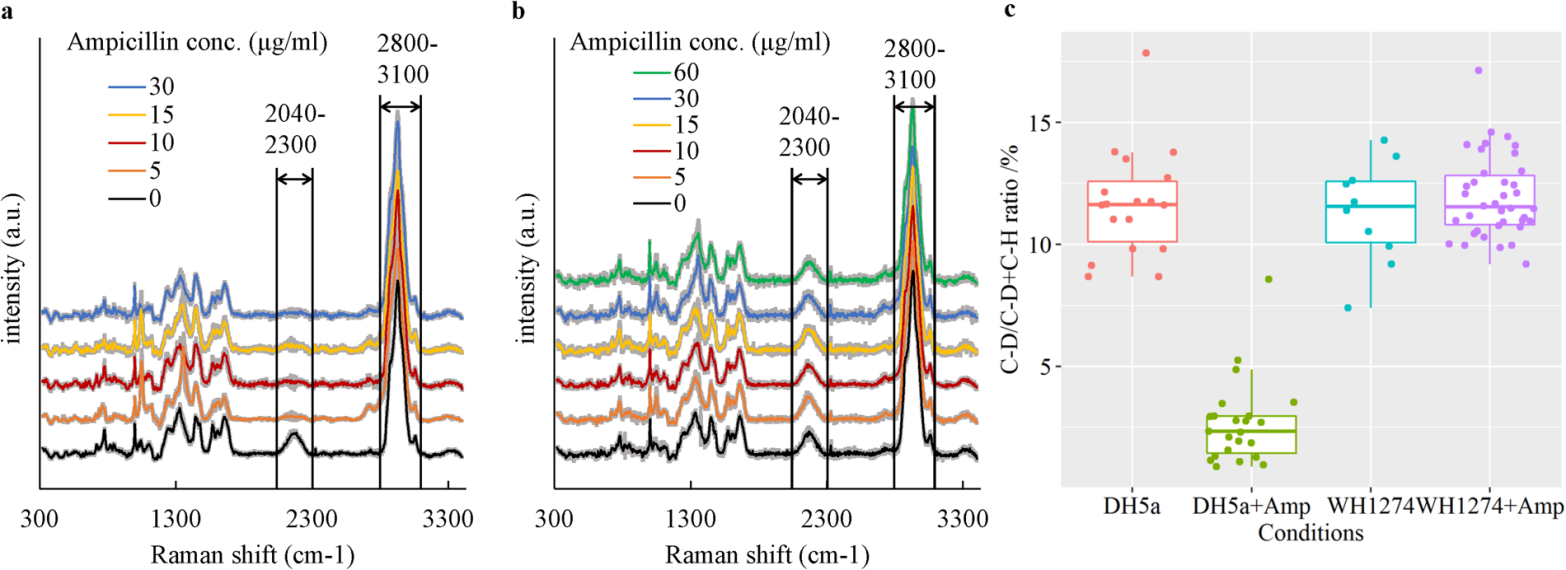
SCRS of *E. coli* after 24-hour incubation in heavy water amended with different concentrations of ampicillin. Each SCRS is averaged with standard deviation. (**a**) Ampicillin susceptible strain *E. coli* DH5α. (**b**) Ampicillin resistant strain *E. coli* WH1274. The lines represent average of SCRS from individual cells and the grey shadow represents standard deviation of SCRS (n = 10 to 46). The bands of C-D (2040 to 2300 cm^−1^) and C-H (2800–3100 cm^−1^) are marked. Spectra were stacked for illustration purpose. (**c**) The C-D ratio of each SCRS obtained for *E. coli* cells after 24-hour incubation in heavy water amended with or without ampicillin. The data for ‘DH5α + Amp’ and ‘WH1274 + Amp’ are combinative data collected form samples with ampicillin concentration of 5, 10, 15, 30 and 60 μg/ml.

SCRS of *E. coli* DH5α and WH1274 (Table 2) were obtained after being incubated in LB medium (v/v 40% D_2_O) with a range of ampicillin concentrations for 24 hours. The SCRS of 10–46 individual cells under each condition are shown in Fig. 1a and b, where each SCRS is averaged with standard deviation. A broad band between 2040 and 2300 cm^−1^ was displayed in SCRS of the susceptible *E. coli* strain DH5α grown in LB with D_2_O (Fig. 1a), which is C-D Raman vibrations shifted from the C-H band at 2800–3100 cm^−1^ ^26^. This C-D band disappeared when ampicillin was present in the culture medium even at concentration as low as 5 µg/ml. The Amp^R^ strain *E. coli* WH1274, in contrast, consistently presented the C-D band in the ampicillin concentration of 0–60 µg/ml (Fig. 1b).

The ratio of C-D/(C-H + C-D) of SCRS under different treatments is plotted in the box charts in Fig. 1c, which shows that the ratio of susceptible *E. coli* DH5a in the absence of ampicillin was significantly higher than those in the presence of ampicillin. This suggests that the metabolic activity of *E. coli* DH5a should be inhibited by ampicillin. The ratio of C-D/(C-H + C-D) dropped from around 12% without ampicillin to 2.5% with ampicillin greater than 5 µg/ml (Fig. 1c). This ratio of Amp^R^ strain *E. coli* WH1274 maintained at 12% in both absence and presence of ampicillin.

**Figure 2:**
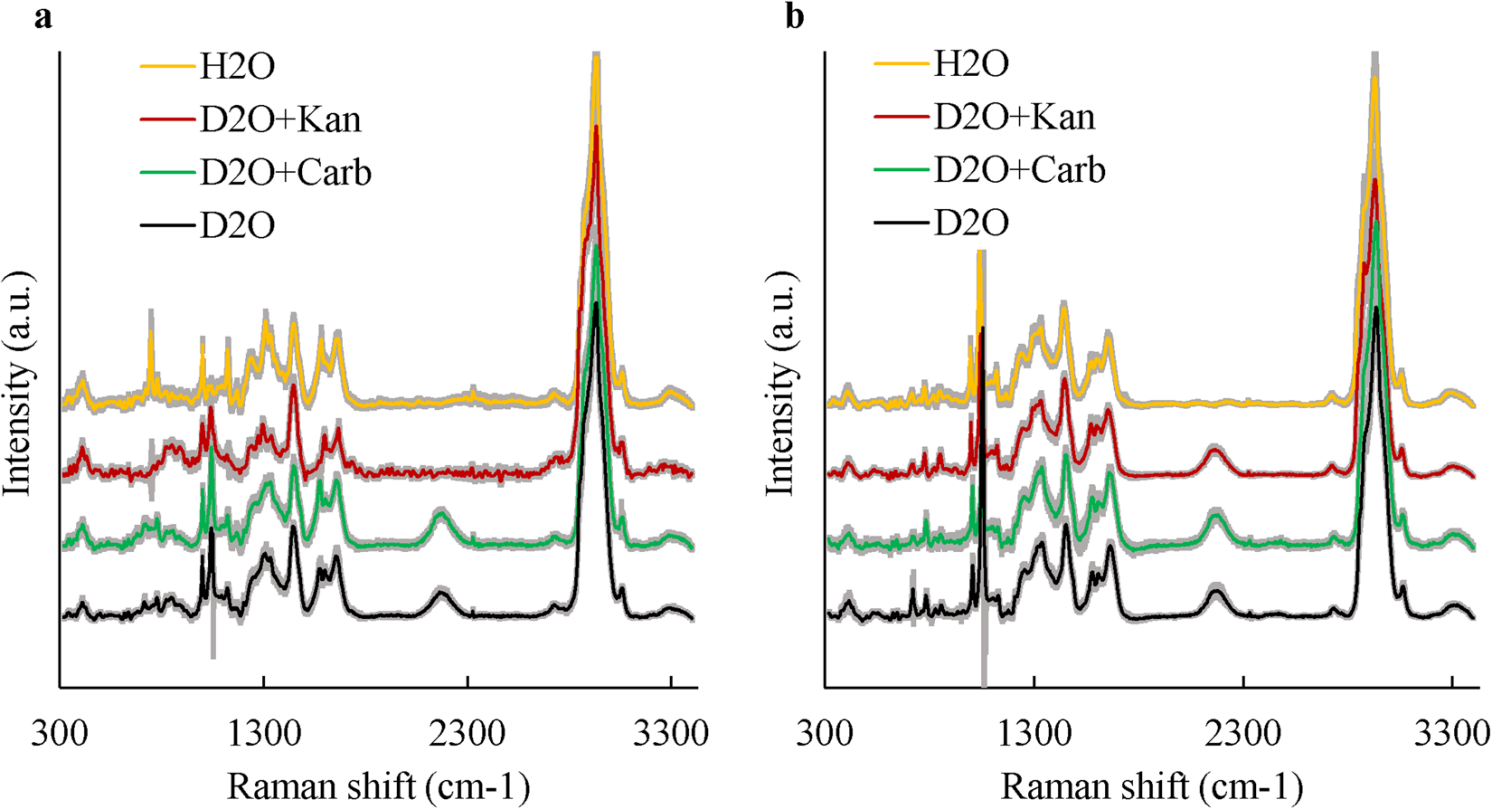
(**a**) SCRS of *Pseudomonas veronii* Carb03 after 24-hour incubation. Each SCRS is averaged with standard deviation. *P. veronii* Carb03 is a carbenicillin resistant isolate from the River Thames in this study. (**b**) SCRS of *Citrobacter freundii* Km03 after 24-hour incubation. *C. freundii* Km03 is a double drug resistant strain isolated from the Thames in this study which can resist kanamycin and carbenicillin. The lines represent average of SCRS from individual cells grey shadow represents standard deviation of SCRS (n = 50). Spectra were stacked for illustration purpose.

Raman-DIP was also applied to environmental isolates to verify its general application. A carbenicillin resistant *Pseudomonas veronii* Carb03 and a kanamycin resistant *Citrobacter freundii* Km03 (Table 2) isolated from the River Thames were also investigated. After 24 hours of incubation in LB- 40% (v/v) D_2_O medium with carbenicillin or kanamycin, 50 SCRS of each treatment of Carb03 and Km03 were obtained and plotted in Fig. 2, with each SCRS averaged with standard deviation. In LB with H_2_O control, the C-D Raman band was absent in SCRS. When *P. veronii* Carb03 were incubated in D_2_O with antibiotics, the C-D peak was exhibited in the presence of carbenicillin but was absent in the presence of kanamycin. This indicates that *P. veronii* Carb03 was Carb^R^ and Kan^S^ (Fig. 2a). Although *C. freundii* Km03 was isolated from LB agar plate with kanamycin, it exhibited C-D band in the presence of both carbenicillin and kanamycin, indicating that *C. freundii* Km03 was not only Kan^R^, but also Carb^R^ (Fig. 2b). The carbenicillin resistance of *C. freundii* Km03 was later confirmed by its growth on LB agar plate with 100 µg/ml carbenicillin.

**Table 2:** Strains used in this study.

Strain	Host	Plasmid	Antibiotic resistance	Reference/origin
DH5α	*Escherichia coli* DH5α	—		
WH1274	*Escherichia coli* DH5α	pWH1274	ampicillin	51,52
Carb03	*Pseudomonas veronii*	—	carbenicillin	Isolated in this study
Km03	*Citrobacter freundii*	—	carbenicillin and kanamycin	Isolated in this study

### Raman-DIP to identify the AMR bacteria in The River Thames water

**Figure 3:**
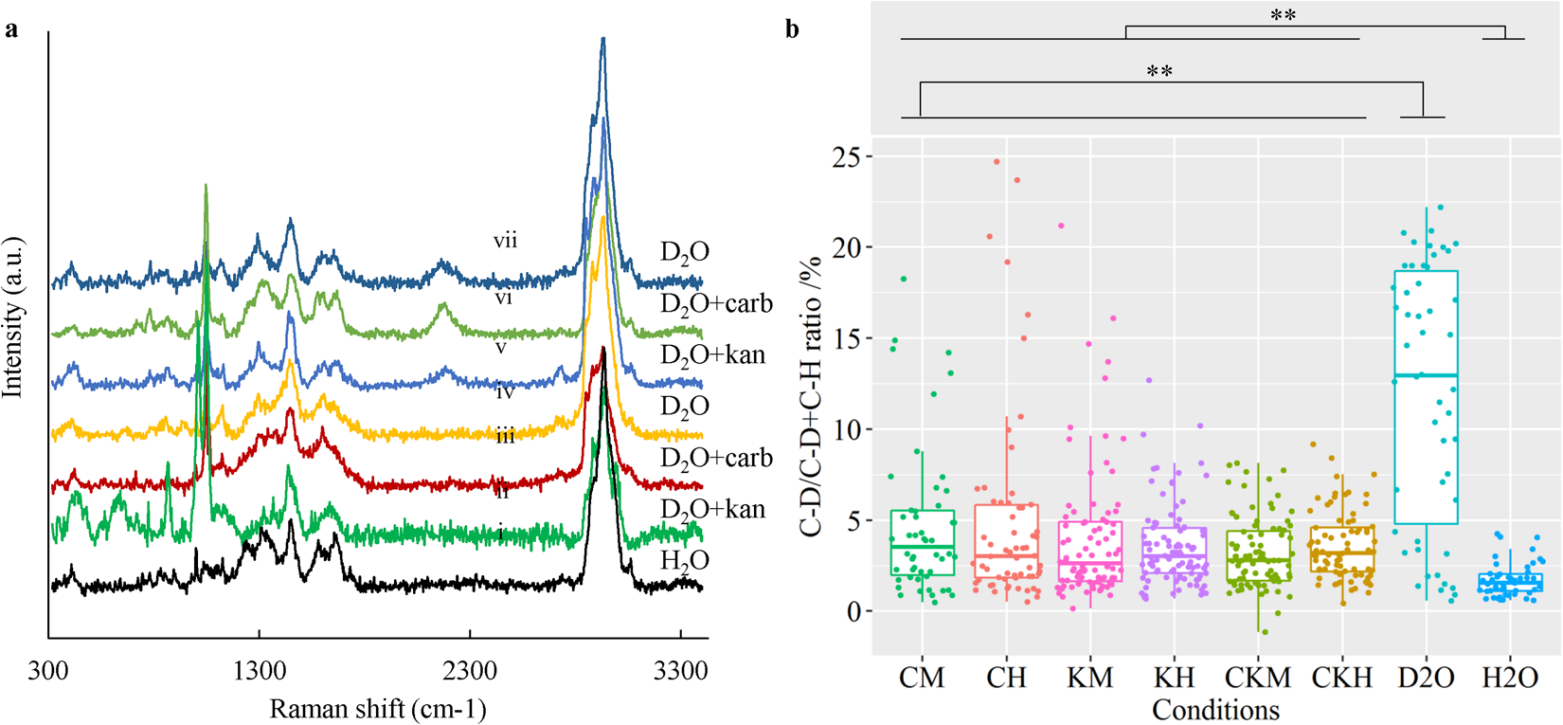
(**a**) Some example of the Raman spectra obtained on the single cell from the River Thames after incubation in amended river water for 24 hours. Carb: carbenicillin; kan: kanamycin. Spectra were stacked for illustration purpose. (**b**) The C-D/C-D + C-H ratio of SCRS for bacterial cells from the Thames sample in Feb 2017 after 24-hour incubation. The replicates in each conditions are 50, 62, 86, 96, 88, 83, 50, 52. Legends: C: carbenicillin; K: kanamycin; M: minimum inhibitory concentration (8 μg/ml for carbenicillin and 2 μg/ml for kanamycin; H: 10 X MIC concentration. The significant difference between the treatment were calculated using student-t test and showed in the figure with double asterisk (p < 0.01). The dashed line represents the selection benchmark of 4.5% (D %) for active bacteria.

The river water samples with a final concentration of D_2_O 40% (v/v) were incubated at room temperature for 24 hours with addition of antibiotics at different concentrations which are listed in Table 3. Figure 3a shows seven typical SCRS with and without C-D in river water as examples. The control sample with water (Fig. 3a–i) did not exhibit a C-D band between 2040 and 2300 cm^−1^. Employing C-D band as a biomarker of general activity, inactive (Fig. 3a-iv) or active (Fig. 3a-vii) cells could be differentiated by adding D_2_O; and in the presence of carbenicillin and kanamycin, inactive (Fig. 3a-ii and iii) or active (Fig. 3a-v and vi) cells can also be distinguished. Around 60–100 SCRS from randomly chosen bacteria were obtained in each treatment of the river sample collected in Feb 2017 and the C-D/(C-H + C-D) ratio was calculated and plotted as box charts shown in Fig. 3b. The box charts of the C-D ratio for the sample collected in Feb 2016 were shown in the supplementary Fig. S2. It shows a large variation in bacterial metabolic activity in the environmental sample with the C-D/(C-H + C-D) ratio in SCRS ranging from 0% to up to 25% (Fig. 3b). The SCRS C-D ratios of the cells exposed to antibiotics were significantly greater (p < 0.01) than the negative control (cells in H_2_O) which suggests the presence of AMR bacteria of single antibiotics carbenicillin and kanamycin resistance or multiple antibiotic resistance (both carbenicillin and kanamycin). The overall C-D ratios of the cells treated with antibiotics were all significantly lower than the cells incubated in the absence of antibiotics (p < 0.01) (Fig. 3b). This implies that most bacteria in the River Thames were antibiotic sensitive or susceptible. Noticeably, no significant differences were found between the samples treated with MIC and those with 10 × MIC concentrations of antibiotics (p < 0.05) (Fig. 3b).

**Table 3:** Combination of antibiotic treatment for the River Thames water sample. Eight and 2 μg/ml are the MIC concentration for carbenicillin and kanamycin.

Condition	D_2_O content (%)	Carbenicillin conc. (μg/ml)	Kanamycin conc. (μg/ml)
1	40	8	0
2	40	80	0
3	40	0	2
4	40	0	20
5	40	8	2
6	40	80	20
7	40	0	0
8	0	0	0

### The isolation of AMR bacteria by Raman activated cell ejection

Raman RACE was applied by ejecting the single cells of whose SCRS contained C-D bands in the presence of carbenicillin or kanamycin. Employing the on-chip whole genome amplification design^27^, multiple displacement amplification reactions from 10 single cells were performed, and the 16 S rRNA was amplified from the genomic DNA of 4 single cells. The 16 S rRNA was blasted with sequences in the NCBI Genbank and the bacterial identification was listed in Table 4. According to the best Genbank blast hit, Carb^R^ Thames_01 was close to human pathogen *Aeromonas veronii* ^28^; Kan^R^ Thames_09 and Thames_10 were close to human pathogen *Stenotrophomonas maltophilia* ^29–32^; and Carb^R^ Thames_11 was an unculturable bacterium (Table 4).

**Table 4:** The identification of RACE isolated AMR bacteria from the Thames water.

	Best hit to Genbank	Resistance	Potential as pathogen	Reference
Thames_01	*Aeromonas veronii* (LC020025.1)	carbenicillin	Human pathogen	28
Thames_09	*Stenotrophomonas maltophilia* (KY078808.1)	kanamycin	Human pathogen	29–32
Thames_10	*Stenotrophomonas maltophilia* (KY078808.1)	kanamycin	Human pathogen	29–32
Thames_11	*Uncultured bacterium clone* (KX626584.1)	carbenicillin		

## Discussion

### C-D band in Raman-DIP indicating metabolic activity of AMR bacteria

This study confirms that Raman-DIP and RACE are effective culture-independent approaches for rapid identification of AMR bacteria at single cell level in environmental samples. This exploits that fact that metabolic activity is in proportional to the level of deuterium from D_2_O taken by the cells^10,25,26^. This process is mediated by bacterial electron carrier NADPH or NADH, which is essential to cell metabolism. NADPH or NADH can exchange H^+^ with D^+^ from D_2_O, and incorporate D^+^ to carbon and form C-D bonds. The incorporation of deuterium into cells causes C-H band at 2800–3100 cm^−1^ shifting to C-D band at 2040–2300 cm^−1^, which is a SCRS ‘silent zone’ and usually free of any Raman signal in most, if not all cells. The ratio of C-D/(C-H + C-D) can be used as a semi-quantitative indicator of general metabolic activity in cells^14,25,26^. It has been previously reported that D_2_O content in growth medium lower than 50% didn’t affect bacterial growth in a broad range of bacteria families^26^, hence 40% D_2_O was used in this study. Figure 1 demonstrates that application of Raman-DIP is able to distinguish the antimicrobial resistant and susceptible *E. coli* strains. Raman-DIP for AMR bacteria was further examined employing two isolates from the River Thames. Carbenicillin, a stable form of ampicillin^33^ was used in river water samples. This result indicates that, after being exposed to carbenicillin, the Carb^R^ *P. veronii* Carb03 remained active and formed C-D band in SCRS. In contrast, Kan^S^ *P. veronii* Carb03 was unable to form a C-D band in the presence of kanamycin (Fig. 2). Interestingly, although *C. freundii* Km03 was originally isolated from LB supplemented with kanamycin, SCRS revealed that this strain was also active in the presence of carbenicillin (Fig. 2b). It suggests that Kan^R^ *C. freundii* Km03 should be also resistant to carbenicillin, which was subsequently verified. The results confirmed that C-D band in SCRS is a sensitive Raman biomarker for detecting AMR bacteria in the presence of antibiotics. And confirmed that Raman-DIP can be used as a universal method for the identification of antimicrobial resistant bacteria.

### Occurrence and abundance of AMR in Thames River

In order to estimate the percentage of metabolically active bacteria in each condition, the mean of the C-D ratio of a negative control plus 3 times the standard deviation, which was calculated to be 4.5%, was set as the baseline indicating cell metabolic activity (Fig. 3b). The C-D ratios for SCRS of i–iv in Fig. 3a are all below 4.5% and that of v–vii are above 4.5%. So single cell a C-D ratio in SCRS greater than 4.5% was considered as active and the percentage of active bacteria was calculated by dividing the active SCRS counts by the total SCRS counts in each treatment.

**Figure 4:**
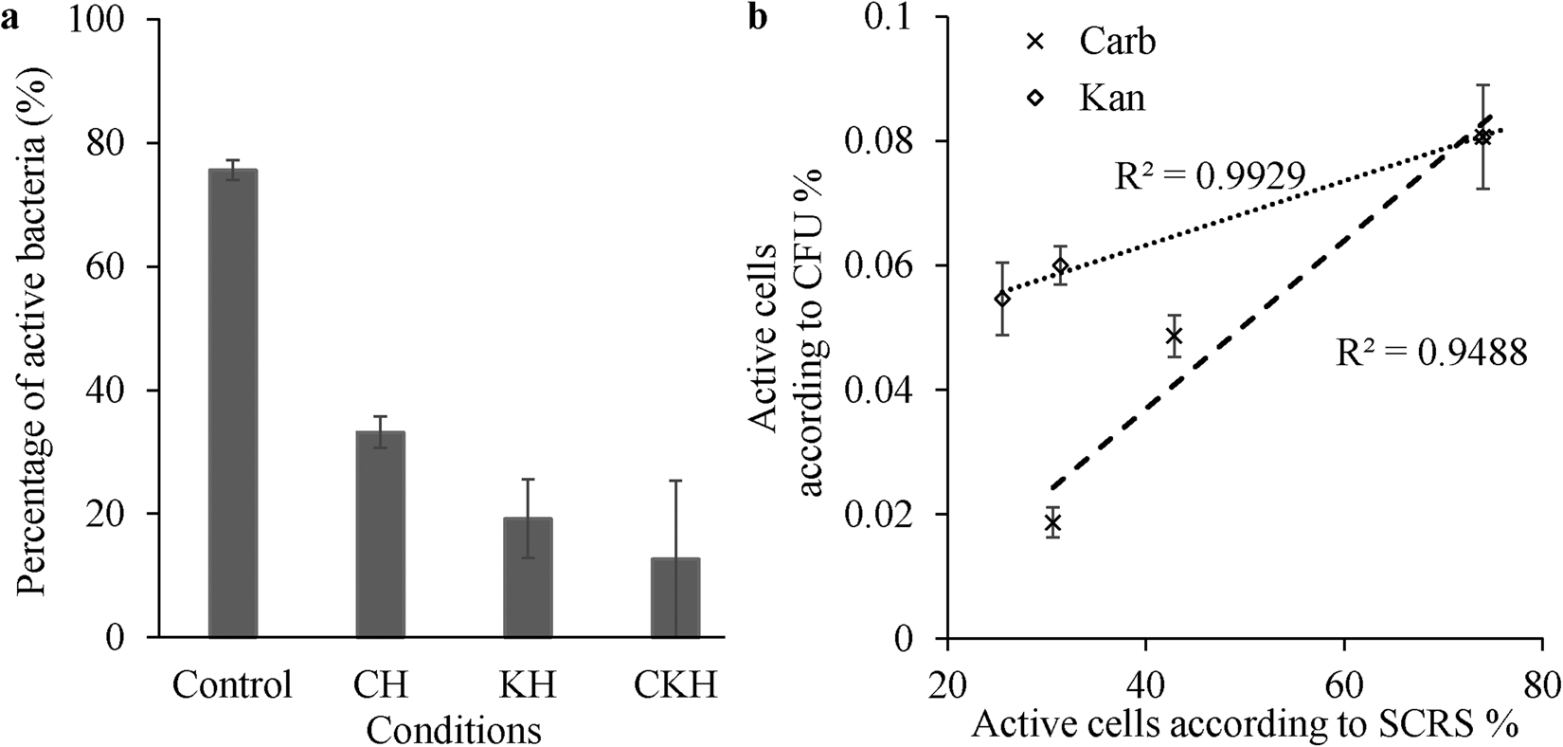
The percentage of active bacteria in the samples from the River Thames revealed by Raman-SIP and the CFU counts obtained by LB agar plate. (**a**) Percentage of deuterium labelled cells revealed by SCRS. Legends: C: carbenicillin; K: kanamycin; H: 10 × MIC concentration. (**b**) The correlation between Raman active cell and CFU counts for cells exposed to Carbenicillin (carb) and Kanamycin (kan). Error bar represents the error for 3 replicates. The R values of linear regression are marked.

The percentage of AMR bacteria in The Thames River water samples is shown in Fig. 4a and Fig. S4. Although the percentages were lower than the non-antibiotic control (76 ± 2%), it indicated a high percentage of natural AMR bacteria in the river: 35 ± 5%, 28 ± 3%, 25 ± 1% bacteria in the samples resistant carbenicillin, kanamycin or both carbenicillin and kanamycin respectively (Fig. 4a). To date, analysis of antimicrobial resistant gene (ARG) has been the only approach available for the investigation of *in-situ* AMR abundance. The ARG abundance of 10^−5^ to 10^−2^ relative to 16S-rRNA reported for river or surface water^34,35^, is at least 10 fold less than detected by Raman-DIP in this case. This implies that in the real environment, the acquisition or exhibition of AMR is largely due to unknown or unspecific mechanisms and beyond our current understanding of ARG.

In this study, the abundance of AMR bacteria employing Raman-DIP was compared to that of conventional cultivation methods. The resistance of 23 and 67% of the culturable bacteria to carbenicillin and kanamycin respectively are comparable to the percentage of ampicillin and kanamycin resistance in river bacteria, which was found to be 59.2% and 25.8% previously reported^36^. The correlation of AMR bacteria abundance revealed by Raman-DIP and the CFU counting was plotted in Fig. 4b. The correlation between Raman-DIP and CFU counts shown in Fig. 4b demonstrates a good agreement with Carb^R^ and Kan^R^ bacteria. It should be borne in mind that the addition of nutrients for cultivation selectively favours for growth of specific populations and thus the real portion of AMR bacteria is unlikely to be identified in their indigenous ecological condition. As adding D_2_O into river water samples had a minimal interference to microbial community, Raman-DIP provides a good approach to identify the AMR bacteria *in-situ*.

We also tried to use river water itself to grow bacteria. The CFU of bacteria grown on agar plates with filter-sterilised river water are shown in Fig. S3. The CFU of river water agar plates were also significantly lower than the microscopic counting (<1%), suggesting that most cells were not recovered by the cultivation method. It also be emphasised that the colonies growing on river water agar plates were very slowly, with an incubation time of at least 6 days. In contrast, the application of Raman-DIP to identify AMR bacteria was performed within 1 day.

The multiple-drug resistance and potential pathogenic risk of the bacteria in the River Thames revealed in this study is concerning (Tables 1 and 4). Among RACE analysis of 4 single cells and 8 isolates, Carb^R^ *Aeromonas sp*. and Kan^R^ *S. maltophilia* were frequently identified species, often associated with human diseases including gastroenteritis and wound infection^28^. Risk factors associated with *Stenotrophomonas* infection include HIV infection, malignancy, cystic fibrosis, neutropenia, mechanical ventilation, central venous catheters^29–32^. Two kanamycin resistant strains were identified as *Citrobacter freundii*, which is responsible for a number of significant infections, including nosocomial infections of the respiratory tract, urinary tract, blood, and many other normally sterile sites in patients^37–40^. Bearing in mind that the vast majority of the microbes are unculturable, the Raman-DIP method can enhance our ability to understand the AMR in natural environment. Various development have been made to integrate Raman spectral acquisition with single cell isolation via optical tweezers or microfluidic devices^19,26,41^. In this work, Raman RACE was applied by ejecting the cells of whose SCRS contain C-D bands in the presence of antibiotics. It has been demonstrated previously that by coupling RACE and MDA, the whole genome of the ejected cells can be recovered in a culture-independent manner^27^. In this study, an unculturable Carb^R^ bacterium was discovered in the water samples from The River Thames (Table 4). This indicated that new AMR bacteria can be revealed by Raman when combined with RACE.

### The dormant majority

The quantification of the fraction actually responsible for bacterial activity is crucial in addressing important ecological questions including carbon/nitrogen cycling and organic matter degradation, and for determining the factors controlling these processes. It is well established that the vast majority of environment microbes are uncultured^42^. Indeed, the cultivation method employing in this study can only recover about 1% of the bacteria in the River Thames (5.0 × 10^6^ cells/ml). By applying a culture-independent approach to the environmental samples, single cell Raman technology enables probing metabolic activity of bacteria in the River Thames without cultivation. Raman-DIP revealed that 76% of the bacteria in the water samples in The River Thames were active according to 400 SCRS profiles from cells randomly chosen from the River Thames sampled in both February of 2016 and 2017 (Fig. 4a), indicating that most bacteria in the River Thames were active. Previously many approaches have been adapted to estimate the active portion of bacteria in river water and sediments, and the active portion was estimated to range from 1.8 to 50% of the total bacteria community and less than 10% in most cases^43–48^. Nevertheless, each approach has their own limitations and bias. Fluorochromes staining is often used to distinguish living and dead cells based on membrane integrity, but membrane integrity is not a sure proof of activity^49,50^. The much higher active percentage of bacteria revealed by Raman-DIP indicates that the activity of a large portion of bacteria could be underestimated using other more established approaches.

The occurrence and spread of antimicrobial resistant bacteria in environment pose a great risk to human health. It demonstrates that Raman-DIP was able to identify and quantify AMR bacteria in natural environment at the single cell level without the need of cultivation. AMR bacteria remain active in the presence of antibiotics, incorporating deuterium from D_2_O, and exhibiting C-D band in SCRS, which is sensitively detectable using Raman-DIP. The approach is highly effective at coupling with RACE, which links the phenotypic function (e.g. antibiotic resistance) and the genotype (single cell genomic DNA). This novel approach has enormous potential for assessing the true extent of AMR not only in the natural environment, but also in human blood, urine and body fluids.

## Method

### Chemicals, microorganisms and growth conditions

All chemicals used in this study were purchased from Sigma-Aldrich unless otherwise stated. Heavy water (99.9% atom %D) and antibiotics solution were filtered through 0.2 μm membrane prior to use. Bacterial strains used in this study are listed in Table 2. *E.coli* strain DH5α employed in this study is susceptible to ampicillin and *E.coli* strain WH1274 derived from DH5a harbouring plasmid pWH1274, conferring on the strain ampicillin resistance^51,52^. Luria-Bertani (LB) broth supplemented with 100 μg/ml ampicillin, 80 μg/ml carbenicillin and 20 μg/ml kanamycin was used for cultivating WH1274, Carb03 and Km03 respectively. Strain DH5α and WH1274 were incubated at 37 °C for 24 h, and strain Carb03 and Km03 were incubated at 28 °C for 24 h.

### Sampling of the river water

The river water sampling was carried out twice on the River Thames in Oxford, England, in February 2016 and 2017 respectively. The coordinates of the sampling site were 51°44′47.0″N, 1°15′21.0″W. The top 5 cm of the surface water was collected in glass bottles and transported to the laboratory at ambient temperature within 30 mins. The temperature of the river water was on both sampling was 5 °C.

### Bacteria counting and isolation

To estimate the total bacterial population in the sample, 1 µl river water was dropped onto slides and dried in the cabinet. The cell number was then counted under 100 × microscope with 8 replicates.

To estimate the cultrable AMR bacteria in the river water samples, 100 μl of the river water was spread onto agar plate containing antibiotics. Two types of agar plates were used. LB agar plate (LBA) and the river water agar plate (RA). The RA plates were prepared by adding agar to autoclaved sterilized river water. The concentration of antibiotics in agar plates were 20 μg/ml for kanamycin and 80 μg/ml for carbenicillin. Colony forming units (CFU) were counted after incubating at 28 °C for 40 hours on LBA plate and 140 hours on RA plate. At least 3 replicates were performed in each condition.

Four of the single colonies on each carbenicillin and kanamycin LB agar plates were isolated and their 16S-rRNA fragments were amplified with 63F (5′-CAGGCCTAACACATGCAAGTC-3′)/1387R (5′-GGGCGGWGTGTACAAGGC-3′) primer pair^53^ (annealing temperature 56 °C) and sent for sequencing (Source Bioscience, Oxford). Two strains Carb03 and Km03 which resisted carbenicillin and kanamycin respectively (see Table 2) were used in the heavy water labelling experiment.

### Heavy water (D_2_O) labelling of the metabolically active bacteria

D_2_O labelling was performed according to previous description^26^ with modifications. D_2_O greater than 50% (v/v) in the medium may inhibit bacterial growth, hence the concentration of 40% D_2_O was used throughout this study for that it was relatively high yet won’t affect the bacteria growth^26^. For pure culture, cells in 5-ml overnight culture of DH5α, WH1274, Carb03 and Km03 were spun down and resuspended to 5 ml LB containing 40% (v/v) D_2_O supplemented with appropriate antibiotics. To study the incorporation of deuterium by the bacteria under different concentration of antibiotics, the concentrations of ampicillin for DH5α and WH1274 were 0, 5, 10, 15, 30 and 60 μg/ml. The concentrations of carbenicillin and kanamycin for Carb03 and Km03 were 80 and 20 μg/ml respectively. *E. coli* strains were kept at 37 °C with 200 rpm for 24 hours. Carb03 and Km03 strains were kept at 28 °C with 200 rpm for 24 hours.

For indigenous bacteria in river, 7 vials of each containing 2.4 ml of the river water mixed with 1.6 ml of D_2_O were prepared (final concentration 40% v/v D_2_O). One vial containing 2.4 ml river water and 1.6 ml H_2_O was set as negative control for the deuterium labelling experiment. The combination of 2 concentrations of carbenicillin and kanamycin was applied to the seven samples containing D_2_O and one control, which is listed at Table 3. The lower concentration in Table 3 was the minimum inhibitory concentration (MIC) for *E.coli* reported in literature^54,55^. The higher concentration was 10 times of MIC. Vials were incubated at room temperature on a multi-well plate shaker with 500 rpm for 24 hours.

### Confocal Raman micro-spectroscopy and spectra processing

Raman micro-spectroscopy was applied to measure general metabolic activity of single cells. Prior to Raman acquisition, cells were washed through centrifugation to remove the medium or impurities in river water which may interfere with observation and Raman detection. Cells were then resuspended in H_2_O and 1 μl of each sample was mounted to a specially treated microscopic slide^27^ and allowed to air dry. The Raman spectra were acquired using a confocal Raman microscope (LabRAM HR Evolution, Horiba Scientific, UK) equipped with an integrated microscope (BX41, Olympus) and a motorised XYZ stage. A 100 × objective (MPIanN, NA 0.9, Olympus) was used to view and acquire Raman signals from single cells. The Raman scattering was excited with a 532-nm neodymium-doped yttrium aluminium garnet (Nd:YAG) laser (Ventus, Laser Quantum, Manchester, UK). Raman measurements with grating 300 l/mm resulted in a spectral resolution of ~2 cm^−1^. The detector was a −70 °C air-cooled charge coupled device detector (Horiba Scientific, UK). The laser spot was located in the centre of individual cell. The laser power on a single cell was about 4.2 mW. Acquisition time for one single cell Raman spectrum (SCRS) was 10 s. The number of the SCRS obtained for pure culture of *E. coli*, *P. veronii*, *C. freundii* and uncultivated the Thames bacteria in each condition were 10 to 46, 50, 50, 50 to 96, respectively.

The baseline of spectra was corrected by Labspec6 software (Horiba Ltd, UK). The intensity of the spectra was normalised by the integration of curve. The peak area assigned to C-D (2040–2300 cm^−1^) and C-H (2800–3100 cm^−1^) was integrated. The ratio of C-D/(C-D + C-H) was calculated to present the extent of deuterium incorporation, which indicates the general metabolic activity of cells.

### Raman activated single cell ejection and single cell genomics

Raman activated cell sorting using single cell ejection technique was performed as described previously^27^. Briefly, 1 μl of deionised water washed cells were mounted to the wells on the sampling chip^27^. The cells with C-D peak presented in SCRS were identified and then ejected into cell lysis buffer in the collection chip by a pulse laser^27^. The single cells were lysed and the whole genome was amplified with Phi29 DNA polymerase on chip. The 16S-rRNA fragment was amplified with 338 F (5′-ACTCCTACGGGAGGCAGC-3′) and 530R (5′-GTATTACCGCGGCTGCTG-3′) primer pair^24^ using the whole genome amplified DNA as the template and sequenced at Source Bioscience, Oxford.

## Electronic supplementary material

Supplementary information
