# Supplementary material for: Raman-Deuterium Isotope Probing for in-situ identification of antimicrobial resistant bacteria in Thames River
Source: Sci Rep. 2017 Nov 30;7:16648. doi: 10.1038/s41598-017-16898-x (PMC5709456; doi:10.1038/s41598-017-16898-x)
Supplement: Supplementary file 1 — Supplementary information [file 41598_2017_16898_MOESM1_ESM.pdf]

Supplementary information

Raman-Deuterium Isotope Probing for *in-situ* identification of antimicrobial resistant bacteria in Thames River

Yizhi Song, Li Cui, José Ángel Siles López, Jiabao Xu, Yong-Guan Zhu, Ian P. Thompson, Wei E. Huang

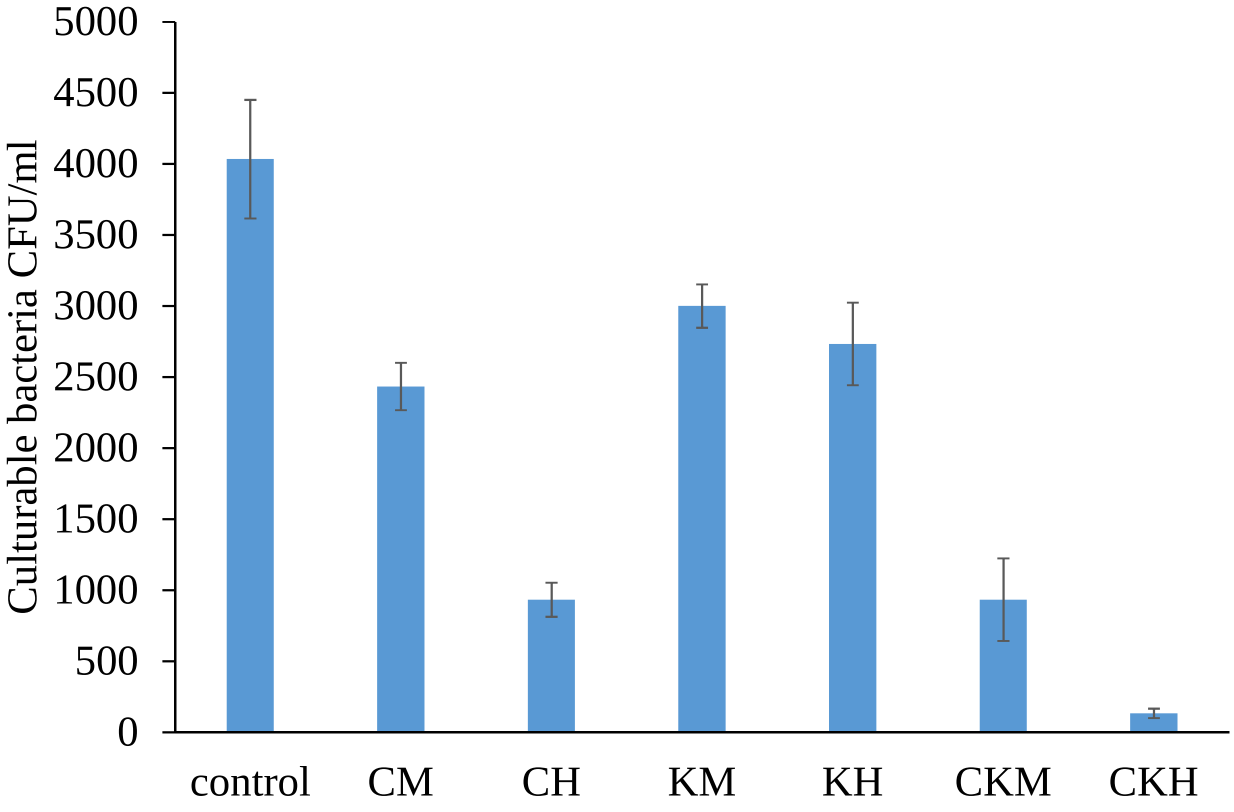

Fig. S1. The percentage of culturable bacteria revealed by CFU counts obtained on LB agar plate for the samples collected in Feb 2017. Legends: control: no antibiotic added; C: carbenicillin; K: kanamycin; M: minimum inhibitory concentration (8 μg/ml for carbenicillin and 2 μg/ml for kanamycin; H: 10 X MIC concentration.

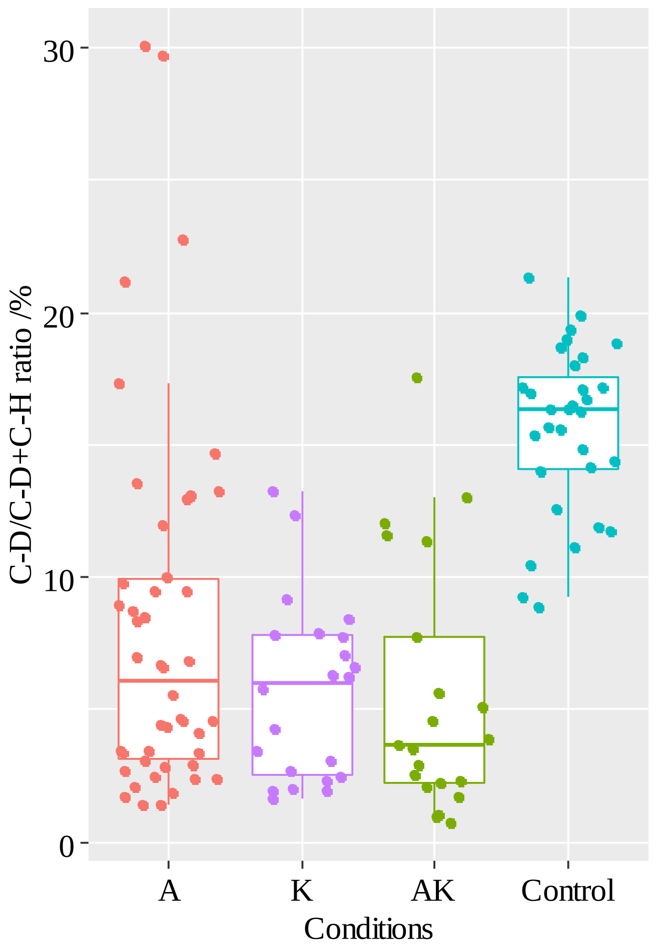

Fig. S2. The C-D/C-D+C-H ratio of SCRS for bacterial cells from Thames River sampled in Feb 2016 after 24 hours incubation. The replicates in each conditions are 46, 22, 21, 31. A: ampicillin; K: kanamycin.

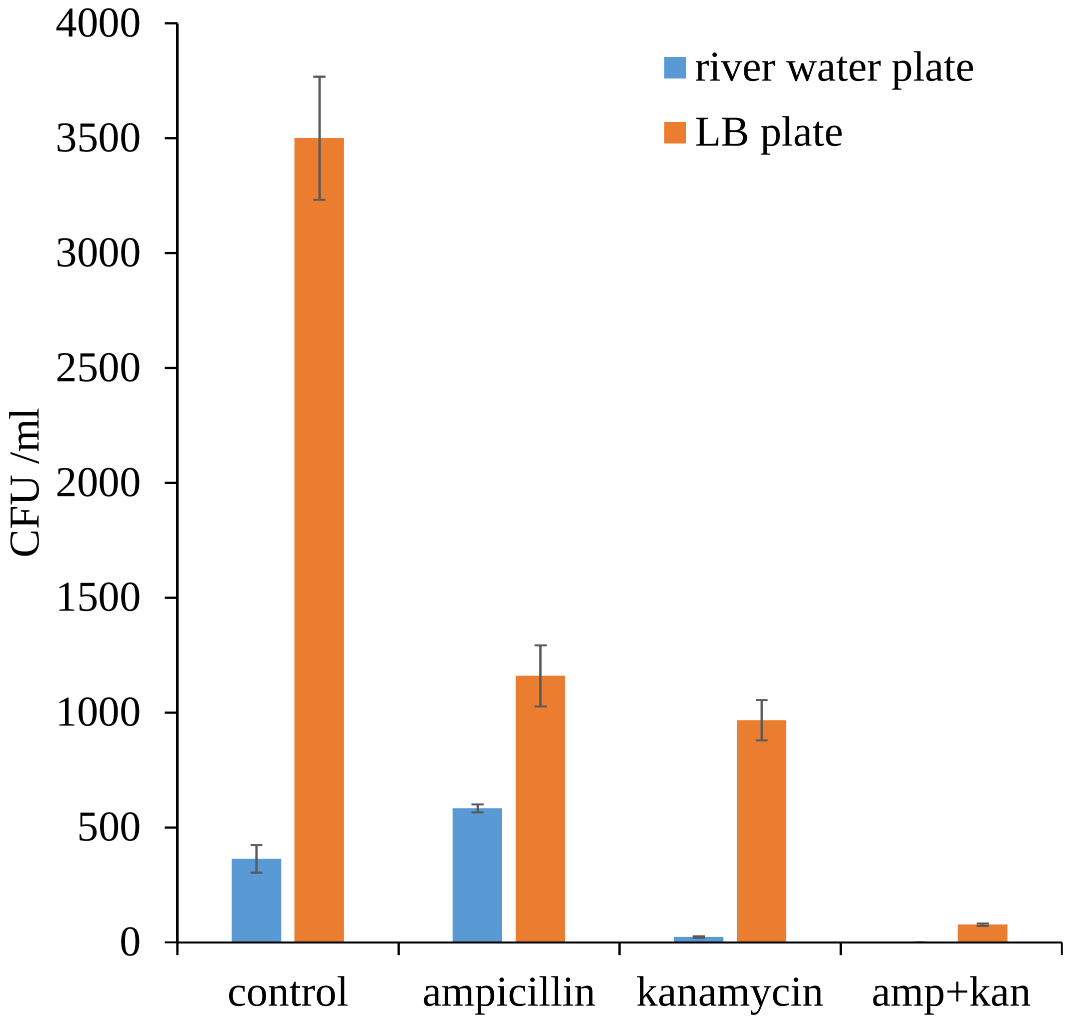

Fig. S3 CFU counting of Thames River sample collected in Feb 2016 by cultivation. The CFU were counted after 140 h incubation on river water plate or 24 h incubation on LB plate.

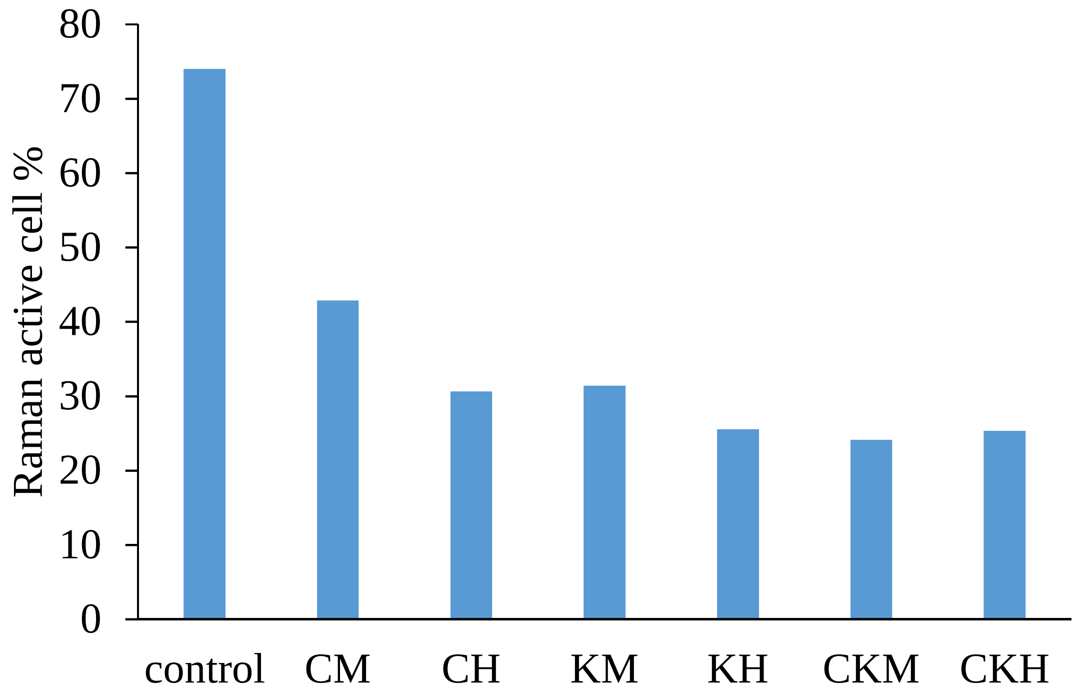

Fig. S4. The percentage of active bacteria revealed by Raman-DIP for sample collected in Feb, 2017. Legends: control: no antibiotic added; C: carbenicillin; K: kanamycin; M: minimum inhibitory concentration (8 μg/ml for carbenicillin and 2 μg/ml for kanamycin; H: 10 X MIC concentration.
